# Altered dynamics of T cell subsets in peripheral blood impacts disease progression in newly diagnosed multiple myeloma

**DOI:** 10.1016/j.bbrep.2025.102104

**Published:** 2025-06-23

**Authors:** Mohini Vig, Lalit Kumar, Shweta Dubey, Ritu Gupta

**Affiliations:** a Amity Institute of Virology & Immunology, Amity University Uttar Pradesh, Noida, India; b Laboratory-Oncology, Dr B.R.A., IRCH, All India Institute of Medical Sciences, New Delhi, India

**Keywords:** Newly diagnosed multiple myeloma, Helper T-cells, Cytotoxic T-cells, Regulatory T-cells, Follicular helper T-cells, T-cell exhaustion

## Abstract

T cells are essential for tumor immunosurveillance and disease regulation in Multiple Myeloma (MM), but their role in disease pathogenesis is not well understood. To investigate this, we analyzed T cell subsets for activation status, relative distribution of naïve and memory T cell populations, regulatory T cells (Tregs), and circulating follicular helper T cells (cT_FH_) in the peripheral blood of 40 newly diagnosed MM (NDMM) patients. We also assessed inhibitory receptor expression CD160, ICOS/CD278, CD152/CTLA-4, and PD-1/CD279 on T cells in peripheral blood. Our results showed reduced T cell numbers, an imbalance between naïve and effector CD4^+^ T cells, and decreased memory Tregs in newly diagnosed MM patients compared to healthy controls. Furthermore, plasma cells in the bone marrow correlated with percentage of activated cT_FH_ cells and inhibitory receptor expressing T cells in peripheral blood indicating that disrupted T cell homeostasis and immune-mediated processes may drive disease progression in NDMM.

## Highlights

• NDMM patients display decreased number of T-cells and altered balance of naïve and memory T cell subsets.
• Disturbed equilibrium of naïve and memory Tregs is a key characteristic of NDMM patients.
• Activated follicular helper T cells correlate with increased bone marrow plasma cells in NDMM.
• Elevated levels of CD160, ICOS, CTLA-4, and PD-1 on T cells, along with their correlation to bone marrow plasma cells, indicate immune-driven disease progression in NDMM.
• These T-cell abnormalities may be considered to harness the benefits of immunotherapy in NDMM.

## 1 Introduction

Multiple myeloma (MM), which manifests as uncontrolled clonal plasma cell proliferation in the bone marrow, is the second most common clonal plasma cell cancer accounting for around 10 % of all hematologic malignancies [1]. Disability-adjusted life Years (DALY) related to MM suggests a correlation between young age (age 15–49 years) and better prognosis in MM [2]. Data from various cancer registries [3] and epidemiology cohorts also indicate increasing incidence and mortality associated with MM [4]. Therefore, MM is a major health concern at the global level; the problem is further compounded by the presence of a rapidly aging population worldwide.

The disease pathogenesis in MM is characterized by immune dysfunction due to the complex interplay between the malignant plasma cells (PC) and immune cells in the tumor microenvironment [5]. It is proposed that aberrant T-cell responses may contribute to the expansion of malignant plasma cell clones in MM and play a critical role in promoting tumor development [6]. Some studies suggest that reduced T cell immunity and T cell immunodeficiency is linked to disease progression in multiple myeloma [7]. There are various proposed mechanisms, including T cell activation, exhaustion, anergy, and senescence, to explain altered T cell immunity in multiple myeloma [[8], [9], [10]] Ineffective antigen processing or presentation *in vivo* by tumor cells or dendritic cells (DC) may also contribute to decreased T cell recognition of tumor cells in multiple myeloma [11]. Overall, T cells are now recognized as an important pathological component in MM. The increased understanding of T cell immune status in hematologic malignancies has led to the development of T cell-specific immune-oncology therapies such as monoclonal antibodies targeting immune checkpoint inhibitors, vaccines, or chimeric antigen receptor (CAR)-T cells [12]. Immune checkpoint inhibitor, Nivolumab, which is a CD279 inhibitor did not produce a favorable therapeutic response in phase I trials which indicate that the spectrum of anti-tumor T cell response in MM still needs to be thoroughly understood [13]. A major drawback of existing studies is the lack of a comprehensive analysis of T cell profiles in MM patients. Therefore, there is an urgent need to assess all aspects of T cell immunity in this disease to guide future immunotherapy. Additionally, T cell profiles and their functional status can serve as biomarkers for disease prognosis or response to treatment in MM.

This study was designed to understand crucial aspects of the T cell response, such as T cell activation, memory pool, regulatory T cells (Tregs), circulating T follicular helper cells (cT_FH_), and expression of inhibitory receptors on T cell subsets to obtain a unified view of the status of the T cell response in newly diagnosed MM (NDMM) patients. Our data suggests an imbalance in peripheral blood T cell subsets at multiple levels, which may provide important cues on restoring T cell response in MM.

## 2 Materials and methods

### 2.1 Patient inclusion criteria

A total of 40 patients newly diagnosed with Multiple myeloma (MM) (males:26, females:14, age range:15–83 years, median age:63 years) attending the myeloma clinic at Institute Rotary Cancer Hospital (IRCH), All India Institute of Medical Sciences, New Delhi were enrolled in this study. The control group comprised of 12 healthy individuals free from any disease/therapy (males:10, females:2, age range: 20–45 years). MM patients were evaluated and diagnosed as per the international myeloma working group criteria [14] and staged based on the International Staging System (ISS) [15], Consensus-based Risk Staging System (CRSS) [16] and Modified Risk Staging System (MRS) [17] (Table 1). Informed consent was obtained from all participants in the study. Ethical approval was obtained from the institutional ethics committee (IEC) at AIIMS, New Delhi (ref number IEC/NP-473/2013).

**Table 1:** Clinical and laboratory characteristics of newly diagnosed multiple myeloma patients.

Characteristics	Newly Diagnosed Multiple myeloma (NDMM) patients (n = 40)
Median age (range)	63 (15–83)
Male (%)	26 (65 %); Comorbidity(1): Scleromyxedema
Female (%)	14 (35 %); Comorbidity(1): Schizophrenia
Past treatment history	Nil
Plasma cell (%)	34.56 ± 31.07
TLC, x10^3^/μL	9.38 ± 6.409
Hb (g/dL)	10.1 ± 2.38
Albumin (g/dL)	3.63 ± 0.79
Calcium (mg/dL)	8.93 ± 1.02
LDH	284 ± 261
Serum beta-2 micro-globulin (mg/L)	10.73 ± 9.05
Creatinine (mg %)	1.81 ± 1.64
M-band Range	No band – 7.3
Kappa (mg/l) Range	9.98–13627.21
Lambda (mg/l) Range	3.6–134408.9
K/L ratio Range	0.00007–1507.43
**ISS Staging**	
Stage 1	4
Stage 2	9
Stage 3	24
Not available	3
**CRSS Staging**	
Stage-1	10
Stage-2	26
Stage-3	3
Not available	1
**MRS Staging**	
Stage-1	7
Stage-2	21
Stage-3	12

### 2.2 Sample preparation and immunophenotyping

Multiparametric immunophenotyping was performed using fluorochrome-conjugated monoclonal antibodies (Table 2) [18,19]. Briefly, blood samples [20,21] were collected in EDTA vials and processed immediately after the collection according to standard protocols (sample preparation, washing, staining and analysis) with specific modifications to preserve the maximum viability of the cells. Data acquisition was performed on a BD FACS Aria III with appropriate compensation controls. For establishing gates and distinguishing negative and positive populations, single-stained and unstained controls were used. Data were analyzed using the built-in analysis tools of Beckman Coulter Kaluza Analysis software version 2.1 to ensure accurate gate placement and appropriate threshold determination. Multiple independent experimental replicates were performed to confirm the reproducibility of gating strategies across different runs, ensuring reliable and consistent analysis.

**Table 2:** Monoclonal Antibodies used for immunophenotyping.

S·NO.	Fluorochrome	Markers
1.	FITC	CD45RA
2.	PE	CD152/CTLA-4
3.	ECD	CD8
4.	PC5.5/PerCP-Cy5.5-A	CD185/CXCR5
5.	PC7/PE Cy7-A	CD62L
6.	APC	CD160
7.	APC700	CD4
8.	APC750	CD3
9.	PB/BV421	CD197/CCR7
10.	KO/BV510	CD223/LAG-3
11.	BV-605	CD25
12.	BV-650	CD279/PD-1
13.	BV-711	CD127
14.	BV-786	CD278/ICOS

### 2.3 Statistical analysis

The differences between patients and healthy controls were compared using the Mann-Whitney *U* Test and values were reported as mean, median, range, standard deviation (SD) and interquartile range (IQR) (Table 3). Correlation analysis was performed using Spearman correlation. All statistical analysis was performed using GraphPad Prism version 10.4.0, with p ≤ 0.05 considered statistically significant.

**Table 3:** The percentage of various T cell subsets in control and NDMM patients determined by flow cytometry analysis is presented as the mean, median, range, standard deviation (SD) and interquartile range (IQR). Statistical significance (p) value was calculated using the Mann-Whitney test. NS: non-significant, ∗p = 0.05, ∗∗p < 0.05

Subsets	Control (N = 12)					NDMM (N = 40)					P-value
	Mean	Median	Range	SD	IQR	Mean	Median	Range	SD	IQR	
CD 3+ T cells	14.83	15.21	2.42–25.02	6.98	9.805	9.49	8.78	0.21–24.91	5.97	8.6	∗
CD4^+^ T cells	54.44	52.78	44.62–77.88	8.27	6.56	48.34	45.58	21.43–83.89	14.91	25.07	NS
CD8^+^ T cells	32.81	35.53	12.94–43.64	8.55	4.56	37.59	39.08	1.17–60.37	13.73	19.83	NS
CD4/CD8 Ratio	1.93	1.52	1.13–6.01	1.32	0.37	2.88	1.18	0.35–53.41	8.30	1.29	NS
T Effector (CD4^+^ CD197- CD127+)	8.45	3.54	0.89–37.39	11.78	6.09	27.95	11.755	3.33–96.36	31.86	39.11	∗
Naive T Cell (CD4^+^ CD197+ CD127+)	82.45	95.34	1.91–99.07	28.14	18.71	58.47	70.92	3.64–98.89	37.07	76.105	∗
Naïve/Effector CD4 Ratio	39.28	23.41	0.546–110.88	40.95	58.77	14.59	4.89	0.03–132.39	26.92	14.48	∗
Naive T cell (CD8^+^ CD197+ CD62L+)	35.63	26.14	13.25–87.9	24.29	27.21	25.37	16.93	0–82.6	22.29	31.02	NS
T Effector (CD8^+^ CD197- CD62L-)	61.16	67.69	12.26–86.86	23.25	23.65	69.30	81.08	1.42–100	27.04	32.25	NS
Naïve/Effector CD8 ratio	1.18	0.35	0.15–7.16	2.008	0.65	1.73	0.22	0–33.24	5.55	0.57	NS
Tregs (CD25^+^ CD127LOW)	6.93	6.15	2.98–13.48	3.00	3.16	7.85	7.82	1.53–27.58	4.20	3.44	NS
Memory Tregs (CD45RA- CD152+)	1.46	1.04	0.08–6.6	1.70	0.88	6.07	2.13	0.13–48.12	10.83	3.34	∗
Naive Tregs (CD45RA + CD152-)	33.98	32.76	19.73–67.98	14.15	16.05	22.66	23.31	4.42–51.09	10.65	12.83	∗
Naïve/Memory Tregs Ratio	57.17	26.29	6.24–251.12	71.05	32.89	26.63	9.51	0.09–262.76	49.55	17.08	∗∗
cTFH (CD45RA- CD185+)	5.89	2.33	0.53–15.68	5.11	7.85	7.02	5.22	1.14–25.69	5.47	7.13	NS
279 + 278+ cTFH (Effector Memory/DP cTFH)	14.67	2.11	1.03–34.12	7.71	14.82	17.33	14.39	0–52.26	12.43	12.31	∗
279- 278- TFH (Central Memory/DN cTFH)	9.97	76.47	0.55–81.86	23.11	58.76	15.18	6.205	0.27–81.09	24.86	57.3825	∗∗
Central Memory/Effector Memory cTFH Ratio	6.82	35.601	0.04–79.47	22.88	92.41	5.30	0.23	0–84.46	16.25	6.23	∗∗
CD278+ CD279- TFH (SP-CD278)	73.53	4.105	0.05–89.68	24.70	31.13	64.09	72.02	2.37–92.26	27.14	63.66	∗
CD279+ CD278- TFH (SP-CD279)	1.83	11.96	0–17.06	4.81	13.94	3.41	0.43	0–36.87	7.94	9.34	∗

### 2.4 Gating strategy/Differentiation of T cells

Sequential gating was used to analyze various T cell subsets. The gating strategy steps are as follows: Discrimination of singlets, viable cells and T cells (Supplementary Fig. 1), T helper cell (CD3^+^CD4^+^) cell subsets (Supplementary Fig. 2), Cytotoxic T (CD3^+^CD8^+^) cell subsets (Supplementary Fig. 3), Analysis of T-regulatory cells (Supplementary Fig. 4) and Analysis of circulating T-follicular helper cells (Supplementary Fig. 5).

## 3 Results

### 3.1 NDMM patients exhibit reduced CD3^+^ T cells and altered homeostasis of naïve and effector T cell subsets

**Fig. 1:**
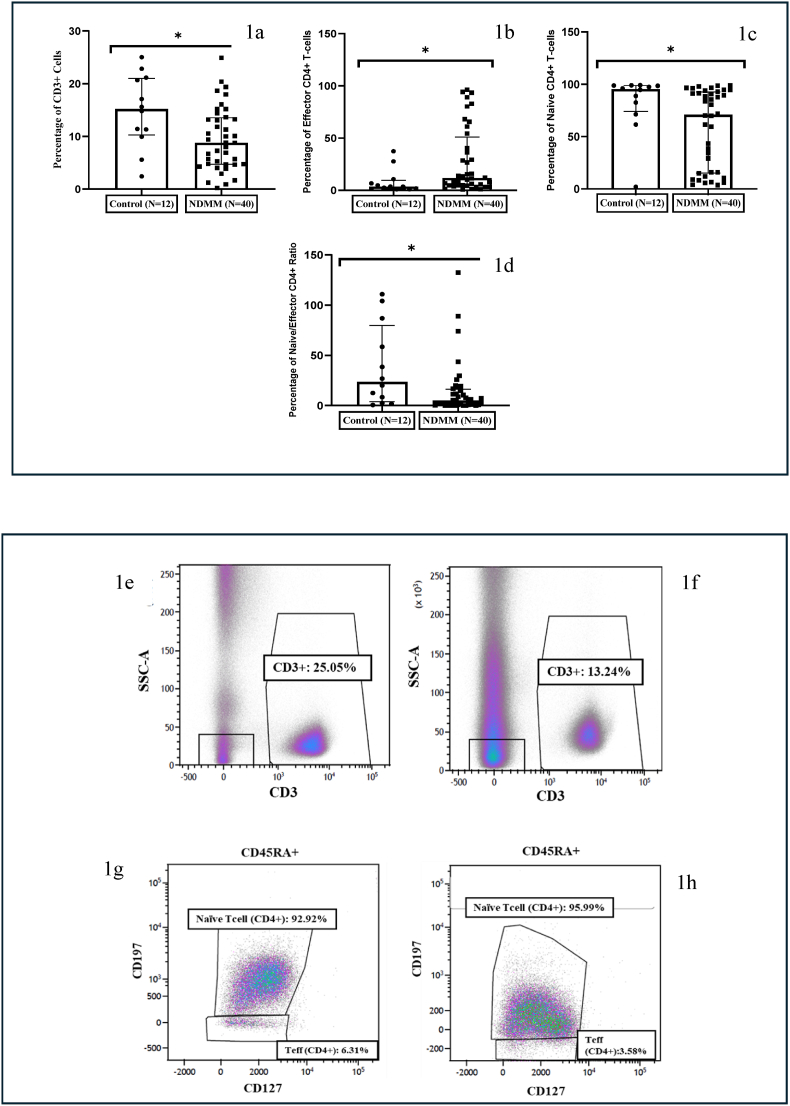
**NDMM patients exhibit reduced CD3^+^ T cells and altered homeostasis of naïve and effector T cell subsets**. (a) Scatter plots with individual points showing significant reduction in the percentage of CD3^+^ T-cells in NDMM group (b) Scatter plots with individual points showing an expansion of Effector CD4^+^ T-cells in NDMM group (c) Scatter plots with individual points showing a reduction in the percentage of naïve CD4^+^ T-cells, (d) Scatter plots with individual points showing a reduction in Naïve/Effector CD4^+^T-cells ratio in NDMM group (e) Density plots of CD3^+^ T-cells from control and (f) NDMM patient (g) Density plots of naïve and effector CD4^+^ T-cells from control and (h) NDMM patient. (The width of the boxes represents the interquartile range, the horizontal line in the boxes represent the median and the whiskers represent the minimum and maximum values. ∗- Significant difference between the groups).

The baseline median absolute counts of CD3 (median 616 cells/mm^3^, IQR: 642.8), CD4 (median 312.7 cells/mm^3^. IQR = 282), and CD8 T cells (223.9 cells/mm^3^, IQR = 266) were low in NDMM patients (Table 4). Analysis of T cell subsets by flow cytometry showed a significant decrease in the percentage of CD3^+^ T cells (p = 0.01); CD3^+^CD4^+^ T Helper cells were reduced and CD3^+^CD8^+^ Cytotoxic T cells were expanded but values were not statistically significant. There was a reduction in the CD4/CD8 ratio in NDMM patients compared to controls, but the differences did not achieve statistical significance (Fig. 1 a, e and f).

**Table 4:** Absolute CD3, CD4, CD8 T-cells and CD4/CD8 ratio counts in NDMM group.

	MEDIAN	MEAN	RANGE	IQR
ABSOLUTE COUNTS CD3 (cells/mm^3^)	619.66	749.12	33.15–1856.4	640.05
ABSOLUTE COUNTS CD4 (cells/mm^3^)	316.35	361.59	8.84–1083.85	268.62
ABSOLUTE COUNTS CD8 (cells/mm^3^)	232.47	287.46	6.18–896.27	266.11
CD4/CD8 RATIO	1.18	1.61	0.35–7.45	1.24

Further, the status of CD4^+^ naïve and memory subsets was assessed based on the expression of CD197 and CD127 markers. The percentage of naïve CD4^+^ T cells defined as (CD4^+^CD197^+^ CD127^+^) was significantly decreased (p = 0.01) in NDMM patients compared to controls. whereas Effector T cell levels (Teff) defined as (CD4^+^CD197^−^CD127^+^) were expanded considerably (p = 0.01) between patients and controls. Central memory (Tcm) (CD4^+^CD197^+^CD127^-^) or effector memory (Tem) (CD4^+^ CD197^-^ CD127^++^) T cell percentages were not different between patients and controls. The naïve/effector CD4^+^ cells ratio also showed a significant decrease (p = 0.01) in NDMM patients compared to controls (Fig. 1 b, c, d, g and h).

Cytotoxic T cells (CD3^+^CD8^+^) were also categorized into 4 subsets based on CD197 and CD62L expression. Naïve (CD8^+^CD197^+^CD62L^+^), effector (CD8^+^CD197^−^CD62L^−^), central memory (CD8^+^CD197^+^CD62L^+^) and effector memory (CD8^+^CD197^−^CD62L^−^). There was no statistically significant difference between naïve or memory CD8 subsets in NDMM patients compared to healthy controls.

### 3.2 NDMM patients exhibit disturbed equilibrium of naïve and memory Tregs

**Fig. 2:**
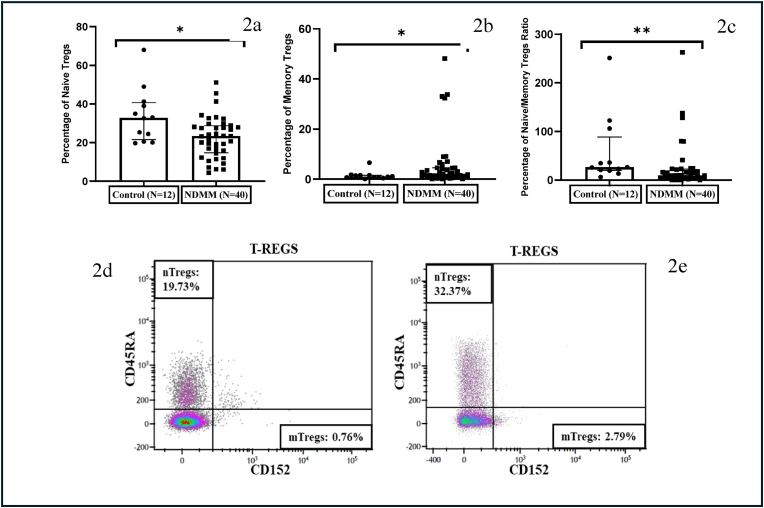
**NDMM patients exhibit disturbed equilibrium of naïve and memory Tregs**. (a) Scatter plots with individual points showing a reduction in the percentage of naive Tregs in NDMM group (b) Scatter plots with individual points showing expansion in memory Tregs in NDMM group (c) Scatter plots with individual points showing a decrease in Naïve/Memory Tregs ratio in NDMM group (d) Density plots of naïve and memory Tregs from control and (e) NDMM patient. (The width of the boxes represents the interquartile range, the horizontal line in the boxes represent the median and the whiskers represent the minimum and maximum values. ∗- Significant difference between the groups).

The levels of peripheral blood regulatory T cells (Tregs) defined as CD3^+^CD4^+^CD25^+^CD127^low^ were comparable between patients and controls. Tregs were also classified into naïve and memory subsets based on CD45RA and CD152 expression, i.e., naïve/resting (CD25^+^CD127^low^CD45RA ^+^ CD152^-^) and effector/memory (CD25^+^CD127^low^CD45RA^−^CD152^+^). The percentage of memory Tregs was significantly increased (p = 0.03) whereas the percentage of naïve Tregs was significantly reduced (p = 0.01) in NDMM patients as compared to controls. The naïve/memory Tregs ratio was also significantly reduced (p < 0.01) in NDMM patients as compared to controls (Fig. 2 a, b, c, d and e).

### 3.3 Circulating T-follicular helper cells in NDMM display features of activation

**Fig. 3:**
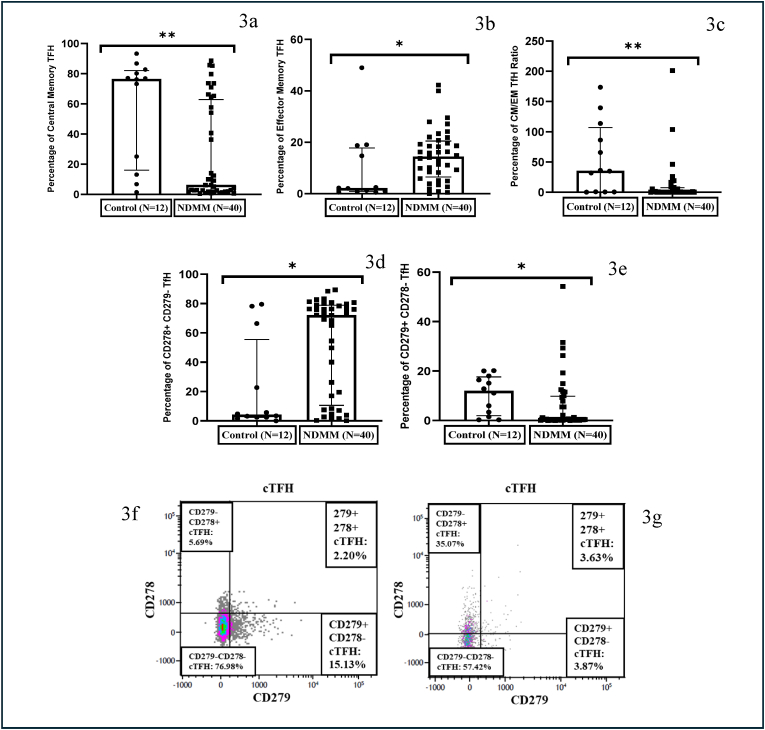
**Circulating T-Follicular Helper cells (cT_FH_ cells) in NDMM display increased expression of CD279 and CD278**. (a) Scatter plots with individual points showing reduction in percentage of central memory cT_FH_ (b) Scatter plots with individual points showing expansion in percentage of effector memory cT_FH_ (c) Scatter plots with individual points showing reduction in Central memory/Effector memory cT_FH_ ratio (d) Scatter plots with individual points showing expansion in the percentage of CD278^+^ CD279^-^ T_FH_ cells (e) Scatter plots with individual points showing reduction in the percentage of CD279^+^ CD278^-^ cT_FH_ cells (f) Density plots of cT_FH_ sub-division from control and (g) NDMM patient. (The width of the boxes represents the interquartile range, the horizontal line in the boxes represent the median and the whiskers represent the minimum and maximum values. ∗- Significant difference between the groups).

Circulating T follicular helper cells (cT_FH_), which are defined as CD4^+^CD45RA^−^ CD185^+^, were comparable between patients and controls. cT_FH_ cells were further analyzed into four subsets based on CD279 and CD278 expression: i) cT_FH_ double positive (DP) for CD279 and CD278 (activated or Effector Memory T_FH_), ii) cT_FH_ subset single positive (SP-CD279) for CD279 and CD278^-^ iii) cT_FH_ subset single positive (SP- CD278) for CD278 and CD279^-^ and iv) a subset double negative (DN) for both CD279 and CD278 (Central Memory T_FH_). The percentage of DN cT_FH_ was significantly decreased (p < 0.01) whereas DP cT_FH_ was increased considerably (p = 0.02) in NDMM patients compared to controls. The central memory/effector memory ratio (p < 0.01) was also significantly decreased in NDMM patients as compared to controls. The SP–CD278 cT_FH_ was significantly increased whereas SP-CD279 cT_FH_ was significantly decreased in NDMM patients compared to controls (Fig. 3 a and b c, d, e, f and g).

### 3.4 Inhibitory receptors expressed on various T cell subsets indicate persistent immune activation in NDMM

**Fig. 4:**
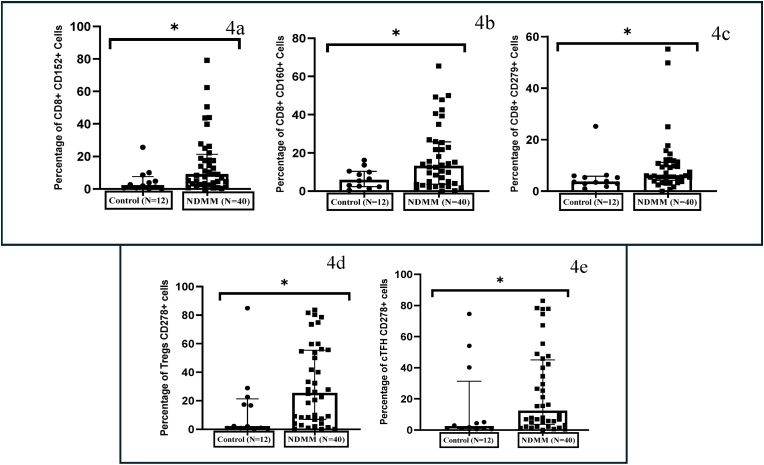
**CD152, CD160 and CD279 are overexpressed only among CD8^+^ T cells in NDMM, whereas CD278 is overexpressed only among Tregs and cTfh subset in the NDMM group**. (a) Scatter plots with individual points showing significant expansion in the expression of CD8^+^ CD152+ cells (b) CD8^+^ CD160+ cells and (c) CD8^+^ CD279+ cells in NDMM group. (d) Scatter plots with individual points showing significant expansion of expression of CD278 on Tregs and (e) cT_FH_ subsets in NDMM group. (The width of the boxes represents the interquartile range, the horizontal line in the boxes represent the median and the whiskers represent the minimum and maximum values. ∗- Significant difference between the groups).

The following inhibitory markers were studied in T cells: CD152 (CTLA-4), CD279 (PD-1), CD160, CD278 (ICOS) and CD223 (LAG-3). Of all the inhibitory markers analyzed, significant differences were noted in the percentage of T cells co-expressing CD152, CD160, CD279 and CD278 between NDMM patients and controls. The proportion of CD8^+^CD152^+^ T cells was significantly increased (p = 0.01) in NDMM patients compared to controls (Fig. 4a). The proportion of CD8^+^CD160^+^ T cells was significantly increased (p = 0.04) in NDMM patients compared to controls (Fig. 4b). The proportion of CD8^+^CD279+ T cells was significantly increased (p = 0.03) in NDMM patients compared to controls (Fig. 4c). The percentage of CD278 co-expressing Tregs or cT_FH_ positive for CD278 cells was significantly increased in the NDMM group compared to controls (Fig. 4d and e). CD223 marker exhibited no significant change in CD4^+^, CD8^+^, Tregs, or cT_FH_ populations between NDMM patients and controls.

### 3.5 Inhibitory receptor expression on CD4 and CD8 T cells increases with the disease stage and correlates with the expansion of plasma cells in the bone marrow

**Fig. 5:**
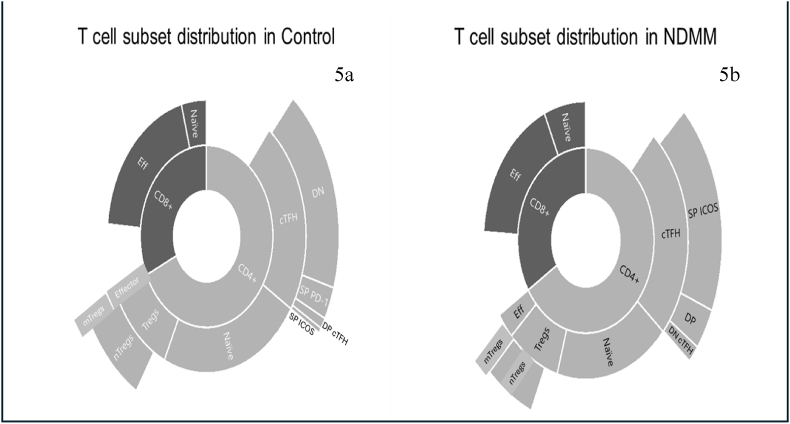
**T cell landscape in NDMM and control**. This sunburst chart shows the percentage subset distribution of various T cell subsets in (a) control and (b) NDMM patients. Changes in naïve and effector CD4 T cells are visible in the chart; NDMM patients exhibit an increase in CD4 T effector cells, an increase in mTregs, and an altered distribution of cTfh subsets. (Teff: effector T cells, Tregs: Regulatory T cells, nTregs: Naïve Regulatory T cells, mTregs: Memory Regulatory T cells, cTfh: circulating T follicular helper cells, DP: circulating T follicular helper cells CD279^+^CD278^+^, DN: circulating T follicular helper cells CD279^−^CD278^-^, SP-CD278: circulating T follicular helper cells single positive for CD278, SP-CD279: circulating T follicular helper cells single positive for CD279).

CD4 and CD8 T cells expressing CD152, CD279, and CD278 expression significantly increased in patients from stage 2 and stage 3 compared to the control group (Table 5). No significant differences were observed in the expression of inhibitory receptors between stage 1 and the control group. The disease stage showed a negative correlation with naïve CD4 T cells (r = −0.4, p = ∗∗, 95 % CI = −0.6117 to −0.1343), naïve/effector CD4 T cell ratio (r = −0.4, p = ∗∗, 95 % CI = −0.6123 to −0.1353), and naïve/memory Tregs ratio (r = −0.3942,p = ∗∗, 95 % CI = −0.6123 to −0.1353) in the peripheral blood. Percent plasma cells (PCs) in the bone marrow of NDMM patients showed a strong positive correlation with CD152 expressing CD8 T cells in peripheral blood, r = 0.45, p = ∗∗∗, 95 % CI = 0.1989 to 0.6517). A moderate level of correlation was also observed between plasma cells in the bone marrow and CD4 expressing CD160 marker (correlation between PCs and Tregs expressing CD160, r = 0.34, p = ∗, 95 % CI = 0.71–0.57; the correlation between percent pcs and CD4 expressing CD160 marker, r = 0.30, p = ∗, 95 % CI = 0.29–0.54). CD279 expression on CD4, CD8, and Tregs was also positively correlated with the percent plasma cells in bone marrow. Double-positive cT_FH_ cells were also positively correlated with PCs in the bone marrow (r = 0.4, p = 0.01, 95 % CI = 0.08286 to 0.5989). The altered subset distribution in NDMM patients is represented in Fig. 5.

**Table 5:** Stage-wise comparison of expression of inhibitory markers on CD4 and CD8 T cells. p value is reported in comparison to the control group. (NS; non-significant).

SUBSETS	STAGE1 (N = 7)	STAGE2 (N = 21)	STAGE3 (N = 12)
CD4^+^ CD152+	NS	NS	∗
CD8^+^ CD152 +	NS	∗	NS
CD4^+^ CD160 +	NS	∗	NS
CD8^+^ CD160 +	NS	∗	NS
CD4^+^ CD223+	NS	NS	NS
CD8^+^ CD223+	NS	NS	NS
CD4^+^ CD279 +	NS	∗∗	NS
CD8^+^ CD279+	NS	∗	∗
CD4^+^ CD278+	NS	∗	NS
CD8^+^ CD278 +	NS	∗	NS

## 4 Discussion

Our data demonstrates some important characteristics of T cell response in newly diagnosed multiple myeloma patients: i) low baseline CD3^+^, CD4^+^, CD8^+^ T cells, and CD4/CD8 ratio. ii) a significantly reduced percentage of naïve cells and Naïve/effector ratio and significantly expanded percentage of effector cells of Th cell lineage iii) a significantly reduced percentage of naive Tregs and naïve/memory Tregs ratio and significantly increased percentage of memory Tregs iv) significantly increased percentage of activated cT_FH_ cells. v) an increased percentage of CD8^+^ T cells expressing inhibitory receptors CD152, CD160 and CD279. vii) an increased percentage of Tregs and cT_FH_ cells expressing inhibitory receptor CD278.

Impairments in T cell numbers and subsets have been previously reported in MM patients. Like our results, low baseline T-cell numbers have been reported in various studies [22,23]. Gu et al. have shown low baseline counts of CD3^+^, CD3^+^CD4^+^ or CD3^+^CD8^+^ subsets in multiple myeloma to be important predictors of survival [24]. We also noted a clear trend of decreasing T cell counts with disease stage in our patient group (data not shown). NDMM patients showed increased presence of naïve Th cells in the periphery and decreased naïve/effector Th cell ratio in the peripheral blood. This indicates an altered T cell homeostasis, in a steady state naïve T cells constitute a predominant proportion of T cell repertoire, however, their reduced percentage in NDMM indicates an ongoing immune activation, antigen-induced engagement of naïve T cells, and possible depletion of naïve T cells by activation-induced cell death or their transition to effector or memory cell type [25]. An increase in Teff cells with increasing malignancy has been reported in MM; Teff cells were observed to be increased in MGUS (monoclonal gammopathy of unknown cause), a state supposed to be the precursor for MM. Changes in T cell dynamics perhaps begin much earlier during the process of disease development in MM [26]; we also observed an increased presence of CD8^+^ T cells expressing inhibitory receptors like CD152, CD160 and CD279 indicating suppression of productive CD8^+^ T cell response in multiple myeloma [27]. Increased expression of inhibitory receptors is also supposed to induce a state of immune exhaustion or anergy in effector Th cells [28]. Increased expression of inhibitory checkpoint molecules like CD279, CD223, CD160, or 2B4, on CD4 and CD8 T cell subsets has been referred to as T cell exhaustion [28,29]. Exhausted T cells exhibit functional characteristics such as loss of effector functions, inert behaviour, and failure to eliminate tumor cells [30]. Cytotoxic T cells rely on helper T cells for optimal tumor elimination. Inadequate help from helper T cells can reduce the cytotoxic activity of CD8^+^ T cells, allowing tumors to evade the immune system [31,32]. Our data suggests that in NDMM patients, Th cells in the peripheral blood undergo chronic immune activation, shifting from active immune surveillance to immune evasion [33].

In our study, differential expression of inhibitory receptors CD279, CD152, CD223, and CD278 on different T cell subsets was observed indicating a lack of functional overlap among inhibitory receptors [34]. There was variation in the expression of the same inhibitory receptor among the NDMM patients analyzed in this study. This may be due to individual-specific anti-tumor immune responses in a particular NDMM patient. Among the inhibitory markers studied on different T cell populations, we observed a significant increase in CD152, CD279, CD160 and CD278 expression on various T cell subsets. Apart from various studies have reported different inhibitory markers in newly diagnosed multiple myeloma, reflecting the complexity of the disease and its impact on the immune system [35]. We observed that T cells express not only a single inhibitory receptor but also co-express many inhibitory receptors (data not shown). This attribute of T cells in the context of tumors is akin to a state of "immune exhaustion"[31]. Commonly studied inhibitory markers in NDMM include CD279, CD152, Tim-3, CD223, TIGIT, CD39, and CD73 [[36], [37]]. The differences in results from various studies may be attributed to disease heterogeneity in NDMM, complexities of the immune microenvironment, and different methodologies used to study these markers on T cells. In our study, the increase in immune exhaustion markers with disease stage and correlation between T cell subsets with plasma cell percentages in bone marrow indicates a clear trend of immune-mediated plasma cell expansion in NDMM. These differences were not immediately apparent when analyzing the overall percentages of T cells. Our data suggests that disease progression in NDMM may be associated with a functional shift in T cell subsets which may require further validation and mechanistic confirmation from functional assays. The limited statistical power of our study coupled with other confounding factors like age, sex, etc may also influence the results. However, significant correlations observed in our study indicate that these are not mere chance events and may have an important role in disease progression which warrants further functional studies.

Tregs are another important T cell subset that can differentiate into effector CD4 T cells and amplify tumor immunosuppression. A combination of Treg-mediated immunosuppression and CD4 effector functions may be a key factor in NDMM as our data suggests an expansion of memory Tregs. The frequency of Tregs did not differ between the NDMM and control group. We also, observed a significant decrease in peripheral blood naïve Tregs in NDMM patients, as compared to controls. Conflicting evidence exists regarding the role of Tregs in MM; most studies suggest an *in vivo* expansion of Tregs in MM [38,39]. In our study, quantification of Tregs was done based on CD4, CD25, and CD127 markers. Functional data from studies has shown that Tregs classified as CD4^+^CD25^+^CD127 ^low/−^ T cells are functionally defined as “real Tregs” [40], we, therefore, used these markers to evaluate the status of Tregs in NDMM and controls. Further analysis of Tregs as naïve Tregs and memory Tregs based on CD45RA, and CD152 expression indicated a significantly reduced ratio of memory to naïve Tregs in NDMM patients as compared to controls indicating a disturbed equilibrium between naïve and memory Tregs in peripheral blood in NDMM. Similar results have been reported in pathological conditions like coronary artery disease [41], chronic obstructive pulmonary disease [42], Alzheimer's disease [43] and multiple sclerosis [44]. In healthy adults, most Tregs present in peripheral blood are memory Tregs which show less suppressive activity than naïve Tregs. Naïve Tregs on the other hand have been shown to exert immunosuppressive function and resistance to activation-induced cell death [45]. Elevated expression of inhibitory markers like CD152 on peripheral blood Tregs of MM patients and enhanced suppressive function of Tregs have been reported in MM [39]. Beyer et al. have reported an *in vivo* peripheral expansion of Tregs from naïve, central memory, and effector memory phenotype; this study utilized CD4, CD25, and Fox-p3 as markers for Tregs and CD45RA and CCR7 to classify Tregs as naïve, central memory or effector memory lineages [46]. The difference in results between our study and the studies mentioned above may be due to differences in markers or patient cohorts included in the study. In multiple myeloma (MM), the intricate bone marrow and peripheral blood microenvironment and the absence of standardized Treg gating strategies have resulted in inconsistent findings regarding their frequency and functional alterations. While reducing Treg numbers or inhibiting their function can enhance anti-tumor immunity, it is essential to balance this approach to prevent immune overactivation and the risk of autoimmunity. [39]. Our data indicates that memory Tregs may be further evaluated for their scope as useful prognostic or therapeutic utility in MM.

The frequency of cT_FH_ cells was not statistically different between the NDMM group and controls, however, marked differences were noted when comparing CD279 and CD278 expression in cT_FH_ cells. The frequency of double-positive cT_FH_ i.e., CD279^+ve^ CD278^+ve^ subsets was significantly increased in the NDMM group as compared to controls. The CD279^+ve^ CD278^+ve^ subset of cT_FH_ cells represents activated cT_FH_ cells which are minimally present in normal individuals but show increased frequency after vaccination [[47], [48], [49]]. In human's majority of cT_FH_ cells express CD279 but no CD278, also referred to as quiescent cT_FH_ [50]. We found a significant positive correlation between double-positive cT_FH_ i.e., CD279^+ve^ CD278^+ve^ cT_FH_ cells and plasma cells in bone marrow. This expansion of DP cT_FH_ cells in NDMM patients may indicate the perpetuation of germinal centre (GC) reaction [48]; We hypothesize from our data that plasma cell expansion in multiple myeloma may be partly driven by the elevation of activated cT_FH_ cells which may further require functional validation. While it is widely known that these CD4^+^ T cells play a critical role in infection and immunization, little is known about their role in cancer. In malignancies originating from T_FH_ cells or B cells, higher T_FH_ cell counts are typically linked to a poor prognosis; in contrast, their presence is generally associated with a better prognosis in a variety of solid organ tumor types that do not originate from lymphocytic cells. A recent study by Nicolas et al. has shown a good predictive value for these cell types in MM and may serve as a therapeutic target or prognostic determinant in MM [51].

Overall, our data suggests a disturbed T cell equilibrium and imbalance in T cell subsets suggestive of persistent immune activation in NDMM patients. An elevated percentage of T cell subsets that had elevated expression of inhibitory receptors such as CD160, CD152, CD279 and CD278, the relative abundance of naïve Tregs and activated cT_FH_ amongst total T cells and correlation with disease stage indicates that a skewed T cell profile may contribute to immune editing and disease development in Multiple myeloma [52] It may be important to conduct a paired analysis of T cell profiling in peripheral blood and bone marrow to identify the impact of T cell abnormalities in NDMM. It may be argued that our data may not directly indicate the role of T cells in the underlying immunopathology of MM as we have not investigated bone marrow. Although analysis of the immune microenvironment in the bone marrow is important for understanding disease regulation in MM, systemic immunity is important for effective immunotherapy [[53], [54], [55], [56]].

## 5 Conclusion

To conclude, T cell homeostasis is disrupted in newly diagnosed multiple myeloma (NDMM). A comprehensive approach is needed to reactivate T cell responses and improve treatment outcomes. Standardizing T cell marker assessments and analyzing immune cell dysfunction in patients before and after therapy will enhance treatment strategies for multiple myeloma.

## CRediT authorship contribution statement

MV: performed experiments, analyzed the data, prepared the initial draft, and edited the manuscript. SD & RG: designed the study, acquired funding, supervised research, analyzed data, and edited the manuscript. RG & LK: patient selection and recruitment. LK: editing and review. All authors reviewed and edited the final manuscript.

## Funding support

The study was supported by an extramural grant funded to RG and SD from the 10.13039/501100001411Indian Council of Medical Research, New Delhi, India; grant number: 5/13/25/2020/NCD-III.

## Declaration of competing interest

The authors declare that they have no known competing financial interests or personal relationships that could have appeared to influence the work reported in this paper.

## Data Availability

Data will be made available on request.
