# Supplementary material for: Altered dynamics of T cell subsets in peripheral blood impacts disease progression in newly diagnosed multiple myeloma
Source: Biochem Biophys Rep. 2025 Jun 23;43:102104. doi: 10.1016/j.bbrep.2025.102104 (PMC12242439; doi:10.1016/j.bbrep.2025.102104)
Supplement: Multimedia component 1 [file mmc1.docx]

**Supplementary Figures:**

**S1**

S1d

**
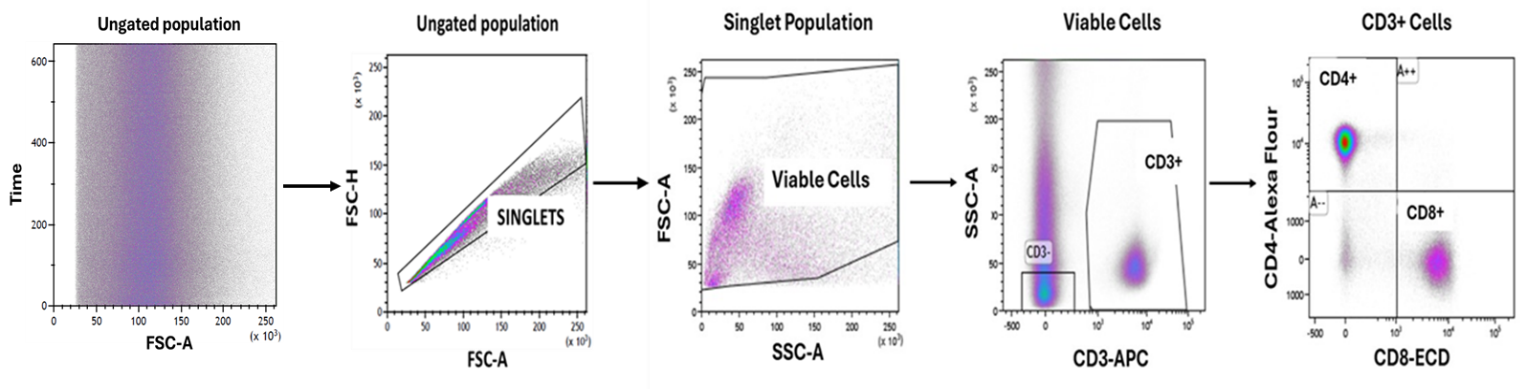
**

S1c

S1b

S1a

**S2**

S2b

S2a


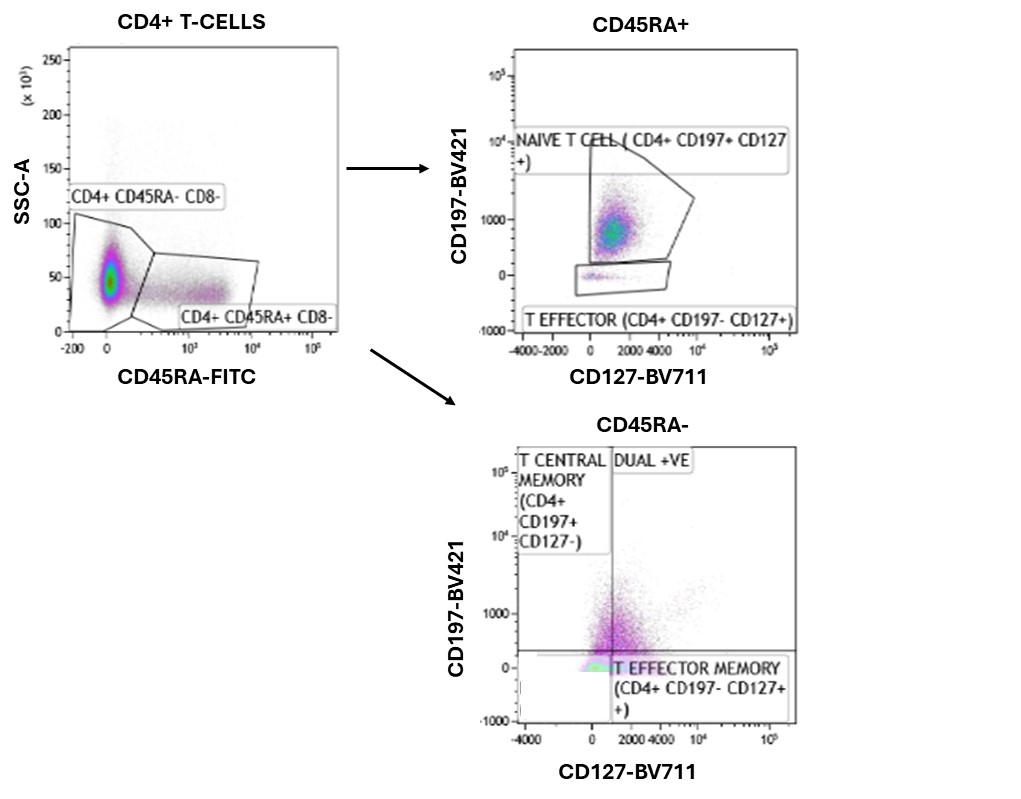


S2c

**S3**

S3bv

S3av


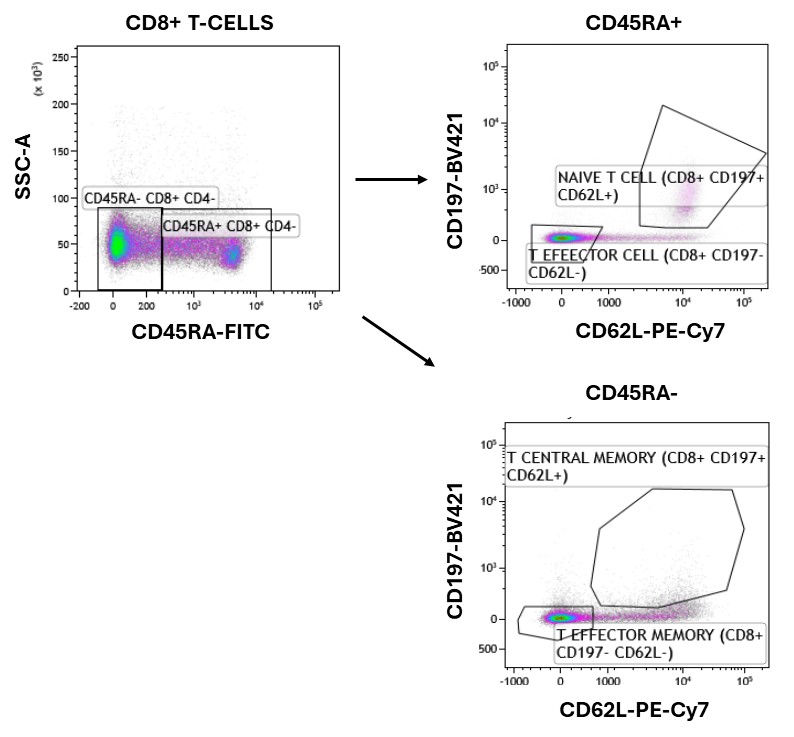


S3cv

**S4**

S4a


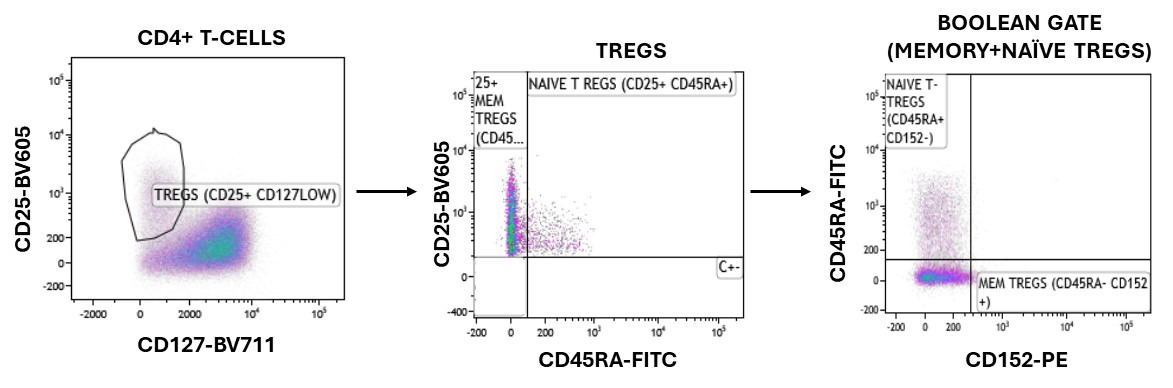


S4c

S4b

**S5**

S5b

S5a


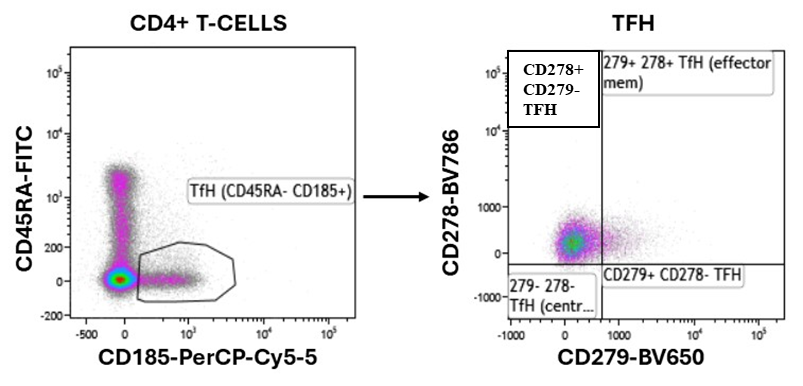


**Legends to Supplementary Figures:**

**Supplementary Figures: Figure S1:** (a) Uniformity of sample checked by plotting Time against FSC-A on ungated population, (b) Discrimination of singlets and exclusion of doublets by plotting FSC-H against FSC-A on ungated population, (c) Discrimination of viable cells by plotting FSC-A against SSC-A on singlet cells, (d) T-cell population (CD3^+^) selected by plotting SSC-A against CD3 on viable cells and (e) CD3^+^ T-cells further divided into CD4^+^ and CD8^+^ T-cells by plotting CD4 against CD8 on CD3^+^ cells.

**Figure S2:** Sub-division of CD4+ T-cells: (a) CD4+ T-cells are further subdivided into two groups based on CD45RA that is CD4^+^CD45RA^-^ and CD4^+^CD45RA^+^. (b) Further, the CD4^+^ CD45RA^+^ population is divided into two subsets i.e. Naïve (CD197^+^CD127^+^) and T-effector (CD197^-^CD127^+^) and (c) CD4^+^CD45RA^-^ the population is also divided into two subsets i.e. T-effector memory (CD197^-^CD127^+^) and T-central memory (CD197^+^CD127^-^) based on CD197 and CD127 expression.

**Figure S3:** Sub-division of CD8+ T-cells: (a) CD8^+^ T-cells are further subdivided into two groups based on CD45RA that is CD8^+^CD45RA^-^ and CD8^+^CD45RA^+^. (b) Further, the CD8^+^ CD45RA^+^ population is divided into two subsets i.e. Naïve (CD197^+^CD62L^+^) and T-effector (CD197^-^CD62L^-^) and (c) CD8^+^CD45RA^-^ the population is also divided into two subsets i.e. T-effector memory (CD197^-^CD62L^-^) and T-central memory (CD197^+^CD62L^+^) based on CD197 and CD62L expression.

**Figure S4:** (a) Analysis of T-regulatory cells (CD4^+^CD25^+^CD127^low^) was done by plotting CD25 against CD127 on CD4^+^ cells. (b) T-regulatory cells are further subdivided into 2 groups i.e., Naïve (CD25^+^CD45RA^+^) and Memory (CD25^+^CD45RA^-^) based on CD25 and CD45RA. (c) Finally, naïve and memory T-regulatory cells are checked for the expression of CD152.

**Figure S5:** (a) Analysis of circulating T follicular helper cells (CD4^+^CD45RA^-^C185^+^) was done by plotting CD45RA against CD185 on CD4^+^ cells. (b) Circulating T Follicular helper cells are further sub-divided into 4 groups i.e., Effector (CD279^+^CD278^+^), Central Memory (CD279^-^ CD278^-^), CD278^+^CD279^-^ and CD279^+^CD278^-^ cT_FH_ based on expression of CD278 and CD279.
